# Low-dose monobutyl phthalate stimulates steroidogenesis through steroidogenic acute regulatory protein regulated by SF-1, GATA-4 and C/EBP-beta in mouse Leydig tumor cells

**DOI:** 10.1186/1477-7827-11-72

**Published:** 2013-07-26

**Authors:** Yanhui Hu, Congcong Dong, Minjian Chen, Jing Lu, Xiumei Han, Lianglin Qiu, Yansu Chen, Jingjing Qin, Xiaocheng Li, Aihua Gu, Yankai Xia, Hong Sun, Zhong Li, Yubang Wang

**Affiliations:** 1 State Key Laboratory of Reproductive Medicine, Institute of Toxicology, School of Public Health, Nanjing Medical University, Nanjing 211166, China; 2 Key Laboratory of Modern Toxicology of Ministry of Education, School of Public Health, Nanjing Medical University, Nanjing 211166, China; 3 Department of Molecular Cell Biology and Toxicology, Jiangsu Key Lab of Cancer Biomarkers, Prevention & Treatment, Cancer Center, School of Public Health, Nanjing Medical University, Nanjing, 211166, China; 4 Department of Nutrition and Food Hygiene, School of Public Health, Nanjing Medical University, Nanjing, 211166, China; 5 Jiangsu Provincial Center for Disease Control and Prevention, Nanjing, 211166, China; 6 Safety Assessment and Research Center for Drug, Pesticide and Veterinary Drug of Jiangsu Province, Nanjing Medical University, Nanjing, 211166, China

**Keywords:** Monobutyl phthalate, Progesterone, Steroidogenic acute regulatory protein, Steroidogenic factors 1, GATA-4, CCAAT/enhancer binding protein-beta, Mouse Leydig tumor cells

## Abstract

**Background:**

The ubiquitous use of dibutyl phthalate (DBP), one of the most widely used plasticizers, results in extensive exposure to humans and the environment. DBP and its major metabolite, monobutyl phthalate (MBP), may alter steroid biosynthesis and their exposure may lead to damage to male reproductive function. Low-doses of DBP/MBP may result in increased steroidogenesis *in vitro* and *in vivo*. However, the mechanisms of possible effects of low-dose MBP on steroidogenesis remain unclear. The aim of present study was to elaborate the role of transcription factors and steroidogenic acute regulatory protein in low-dose MBP-induced distruption of steroidogenesis in mouse Leydig tumor cells (MLTC-1 cells).

**Methods:**

In the present study, MLTC-1 cells were cultured in RPMI 1640 medium supplemented with 2 g/L sodium bicarbonate. Progesterone level was examined by I125-pregesterone Coat-A-Count radioimmunoassay (RIA) kits. mRNA and protein levels were assessed by reverse transcription-polymerase chain reaction (RT-PCR) and western blot, respectively. DNA-binding of several transcription factors was examined by electrophoretic mobility shift assay (EMSA).

**Results:**

In this study, various doses of MBP (0, 10(−9), 10(−8), 10(−7), or 10(−6) M) were added to the medium followed by stimulation of MLTC-1 cells with human chorionic gonadotrophin (hCG). The results showed that MBP increased progesterone production and steroidogenic acute regulatory protein (StAR) mRNA and protein levels. However, the protein levels of cytochrome P450scc and 3 beta-hydroxy-steroid dehydrogenase (3 beta-HSD) were unchanged after MBP treatment. EMSA assay showed that DNA-binding of steroidogenic factors 1(SF-1), GATA-4 and CCAAT/enhancer binding protein-beta (C/EBP-beta) was increased in a dose-dependent manner after MBP exposure. Western blot tests were next employed and confirmed that the protein levels of SF-1, GATA-4 and C/EBP-beta were also increased. Additionally, western blot tests confirmed the expression of DAX-1, negative factor of SF-1, was dose-dependently down regulated after MBP exposure, which further confirmed the role of SF-1 in MBP-stimulated steroid biosynthesis.

**Conclusions:**

In conclusion, we firstly delineated the regulation of StAR by transcription factors including SF-1, GATA-4 and C/EBP-beta maybe critical mechanism involved in low-dose MBP-stimulated steroidogenesis.

## Background

Phthalates (PAEs) are ubiquitous in environmental media and human bodies [1]. They are used as plasticizers in polyvinyl chloride plastics that make consumer products such as flexible plastic and toys, curtains, wallpaper, food packaging, plastic wrap and medical devices [2]. PAEs can diffuse to the environment easily, and humans are exposed to PAEs through ingestion, inhalation, and dermal exposure [3].

Dibutyl phthalate (DBP) is one of the most commonly used PAEs in polyvinyl chloride products, which is widely detectable in milk, blood and urine in the general population [4,5]. DBP may exert adverse anti-androgenic effects and its exposure may lead to damage to male reproductive function, such as morphological changes in sperm, shortened anogenital distance (AGD) which is a marker of feminisation in male infants [6-8]. Furthermore, previous studies revealed that administration of DBP to male mammals during sexual differentiation resulted in malformations in male offspring including altered testicular weight, and altered testosterone levels [9-11]. DBP and its active metabolite, monobutyl phthalate (MBP), have been proven to have no androgen receptor binding in transcritional activation assays [12]. So, DBP/MBP may cause adverse anti-androgenic effects though impairing testosterone synthesis [13,14]. Whereas these results revealed the important effects of DBP/MBP on steroidogenesis, all these results were obtained from high administrations of DBP or MBP, usually 500 mg/kg body weight per day or more which were higher than the published data of human exposure (maximal level: 113 μg/kg body weight per day) [15,16].

It is estimated that human exposure to DBP ranging from 0.84 to 113 μg/kg body weight per day [15]. Thus, chronic exposure of humans to low doses of DBP is ubiquitous. Some studies have focused on the effect of low-dose of DBP/MBP on steroidogenesis (Fan J, et al. 2010. Endocrinology 151 3348–62) [17,18]. In these studies, relatively low doses of DBP/MBP resulted in increased steroidogenesis *in vitro* and *in vivo*. Nonetheless, the underlying mechanisms of possible effects of low-dose MBP on steroidogenesis remain unclear.

In the steroid biosynthesis pathway, steroidogenic acute regulatory protein (StAR) is a key protein and plays an essential role in the delivery of cholesterol to mitochondria which is considered as the rate-limiting step in steroid biosynthesis [19,20]. Moreover, previous studies also indicates that transcription factors exert important roles in regulation of StAR expression, including steroidogenic factors 1 (SF-1) [21-23], CCAAT/enhancer binding protein-beta (C/EBP-beta) [24,25], and GATA-4 [23,25].

The cell line of mouse Leydig tumor cells (MLTC-1 cells) is an established mouse Leydig cell line, and is able to synthesize progesterone as the major steroid in response to human chorionic gonadotrophin (hCG). The MLTC-1 cell line also keeps the consistent gonadotropin-response compared with the primary Leydig cell line before progesterone synthesis. Therefore, in this study, in order to provide mechanistic insight into low-dose MBP-induced distruption of steroidogenesis, we focused on expression of StAR and the regulation of transcription factors including SF-1, GATA-4 and C/EBP-beta on StAR expression after MBP treatments *in vitro* using MLTC-1 cells.

## Methods

### Chemicals and reagents

MBP was purchased from Tokyo Kasei Kogyo Co., Ltd. (Tokyo, Japan). Human Chorionic Gonadotropin (hCG), bovine serum albumin (BSA), dimethyl sulfoxide (DMSO), sodium dodecyl sulfate (SDS), diethylpyrocarbonate (DEPC), and 3-(4,5-dimethylthiazol-2-yl)-2,5-diphenyl tetrazolium bromide (MTT) were obtained from Sigma-Aldrich (St. Louis, MO, USA). Fetal bovine serum (FBS), RPMI 1640 medium, streptomycin sulfate, antibiotic penicillin G sodium (10,000 U/mL) and phosphate-buffered saline with Ca^2+^ and Mg^2+^ (PBS+) were obtained from Gibco BRL (Grand Island, NY, USA). Reagents for real-time reverse transcription polymerase chain reaction (RT-PCR) were obtained from Invitrogen Life Technologies (Carlsbad, CA). All other chemicals used were of analytical grade.

### Cell culture and treatments

MLTC-1 cells were obtained from Cell Institute of Shanghai, Chinese Academy of Sciences (Shanghai, China) and maintained in RPMI 1640 medium supplemented with 2 g/L sodium bicarbonate, 100 U/mL penicillin, 100 μg/mL streptomycin sulfate, and 10% fetal bovine serum (FBS, pH 7.2). Cells were cultured in a humidified atmosphere containing 95% air and 5% CO_2_ at 37°C. Medium changes and cell subculture were conducted every 1 or 2 days.

MLTC-1 cells were seeded into 24-well plates at concentration of 1×10^5^ cells/mL in 1 mL of medium per well for progesterone measurement as this cell line produces progesterone as the major steroid in response to trophic hormones [26]. After preincubated for 24 h, the medium was removed and the cells were washed twice with PBS+. MBP was firstly dissolved in DMSO and then adjusted to a concentration of 10^-3^ M with serum-free RPMI 1640 medium as stock solution. Cells were treated with MBP at concentrations of 0, 10(−9), 10(−8), 10(−7), or 10(−6) M or its solvent DMSO for 24 h. The final concentration of DMSO was 0.1%. Then, cells were washed with PBS+ and serum-free medium (SFM). Subsequently, cells were stimulated for 4 h with hCG in SFM supplemented by 0.1% BSA. The medium was collected for progesterone determination and the cells were either dissolved in lysis buffer containing the protease inhibitor phenylmethylsulfonyl fluoride for protein determination or prepared for other biochemical assays.

### Cell viability assay

Cell viability was evaluated by the MTT proliferation assay. MTT (5 mg/mL) was sterilized by filtration through a 0.22 μM Millipore® filter and stored at 4°C. Cells were plated at a density of 1.5×10^4^ per well in 96-well plates. After 24 h of incubation in the different doses of MBP, 25 μl MTT was added to each well and the cells were incubated for 4 h at 37°C. Non-treated cells served as a negative control. The medium was removed and 150 μl DMSO was added to each well and oscillation was then performed for 10 min. The absorbance was determined at 490 nm. Results were presented as percentage of the values measured in untreated control cells.

### Detection of progesterone

Progesterone in the medium was measured with I^125^-pregesterone Coat-A-Count radioimmunoassay (RIA) kits (Beijing North Institute of Biological Technology, China). The minimum detectable concentration of progesterone was 0.2 ng/mL. Inter-and intra-assay coefficients of variation were <10% and <15%, respectively. Cellular protein levels were quantified using the BCA Kit (Beyotime Institute of Biotechnology, Shanghai, China).

### Quantitative real-time RT-PCR assay

Cells were plated at a density of 1 × 10^6^ per well in 6-well plates (Corning®Gorilla® Glass) with a volume of 3 mL per well and treated with MBP for 24 h, then stimulated by hCG for 4 h. To measure the level of StAR mRNA expression in MLTC-1 cells, total RNA was isolated using Tripure Isolation Reagent. Total RNA (2 μg) was converted to cDNA using PrimeScipt® RT Master Mix Perfect Real Time Kit from Takara Biotechnology Co. Ltd (Dalian, China). The resulting cDNA was used as a template for real-time reverse transcription-polymerase chain reaction (RT-PCR). The primers used were as follows: rodent glyceraldehyde-3-phosphate dehydrogenase (GAPDH), sense 5’-GCCGGTGCTGAGTATGTC-3’ and antisense 5’-CTTCTGGGTGGCAGTGAT-3′ spanning from 311 to 601, 291 bp; StAR, sense 5’-ATCATTGTGCCGACTTCCCTAC-3′ and antisense 5’-ACCAGGTTAGCCTCAGTATTAGA-3’ spanning from 1386 to 1671, 286 bp. The values were normalized using GAPDH as the internal standard. All reactions were performed on an ABI PRISM 7900HT Sequence Detection System using SYBR Green PCR.

### Protein isolation and western blot analysis

Concentrations of protein were quantified by the BCA protein assay kit. Samples were used immediately or stored in aliquots at −80°C until use. Extracts were thawed only one time prior to use. Total proteins (80 μg) solubilized in the sample buffer (25 mM Tris, pH 6.8, 1% SDS [w/v], 5% beta-mercaptoethanol [v/v], 1 mM ethylene diaminetetraacetic acid [EDTA], 4% glycerol, and 0.01% bromophenol blue) were loaded onto a 12.3% SDS (w/v) polyacrylamide gel electrophoresis (SDS PAGE; Mini-Protean II, Bio-Rad Laboratories, Hercules, CA). SDS-PAGE was performed at 60 V for 1 h and then at 100 V for 2 h using standard running buffer (24 mM Tris, 0.19 M glycine, and 0.5% SDS, pH 8.3) as described previously with minor modifications (Manna et al., 2004). The proteins were then electrophoretically transferred to a polyvinyldiene difluoride membrane (PVDF, Bio-Rad, Hercules, CA) at 0.15A for 1–1.5 h at room temperature with transfer buffer (20 mMTris, 150 mM glycine, 10% methanol, and 0.01% SDS). The membranes were incubated in blocking buffer (Tris-buffered saline, TBS containing 0.2% Tween-20 and 5% non-fat milk) for 2 h at room temperature, followed by incubation overnight at 4°C with specific mouse polyclonal antibodies for StAR (Santa Cruz, CA, USA, 1:100 dilution), P450scc (Beijing Biosynthesis Biotechnology Co., LTD, 1:500 dilution), 3 beta-HSD (Santa Cruz, CA, USA, 1:200 dilution), SF-1 (Santa Cruz sc-28740x, 1:100 dilution), DAX-1 (Santa Cruz sc-13064, 1:100 dilution), C/EBP-beta (Santa Cruz sc-150x, 1:100 dilution), or GATA-4 (Santa Cruz sc-1237x, 1:100 dilution). The membranes were washed three times (10 min each) in TBS buffer and incubated for 1 h at room temperature followed by incubation with goat anti-mouse, goat anti-rabbit or rabbit anti-goat secondary antibody conjugated with horseradish peroxidase at 1:1000, and the specific signals were detected by chemiluminescence with ECL Western blotting detection kit (Amersham Life Science Limited) after a final 1–1.5 h wash in TBST. After exposed to x-ray films for 1–3 min, the immunospecific bands were quantified. Densitometry was performed using ImageJ software. All blots were normalized to GAPDH and expressed as fold change over the control group.

### Electrophoretic mobility shift assay

Oligonucleotide sequences of SF-1, GATA-4, and C/EBP-beta EMSA were used: SF-1 (−56/-30 bp), 5’Bio-TGATGATGCACAGCCTTCCACGGGAAG-3’; GATA-4 (−76/-51 bp), 5’Bio-GATGACTTTTTTATCTCAAGTGATGA-3’; C/EBP-beta (−125/-100 bp), 5’Bio-ACTGCAGGATGAGGCAATCATTCCAT-3’. Electrophoretic mobility shift assay (EMSA) was performed for analysis of DNA-binding of SF-1 according to the manufacturer’s protocol using the Pierce Light Shift Chemiluminescent EMSA kit. Briefly, 10 μg protein nuclear extracts were obtained by Nuclear and Cytoplasmic Protein Extraction Kit. Nuclear extracts from nontreated or MBP-treated (24 h) MLTC-1 cells were incubated with double-stranded binding fragments at room temperature for 20 min. After incubation, samples were separated on a 0.5×TBE SDS-PAGE minigel and electrophoretically transferred to a nylon membrane. After exposed to x-ray films for 1–5 min, relative intensity of the shifted band was normalized by the value from the control group. EMSA for measuring DNA-binding of GATA-4, and C/EBP-beta was conducted in the same manner as that applied to the SF-1 DNA-binding assay. Densitometry for shifted bands was determined by ImageJ software.

### Statistics analysis

Data were expressed as mean ± S.E. for all experiments done three times in quadruplicate for each treatment. Statistical differences between the treatments and the control were determined by one-way analysis of variance (ANOVA) and least significant difference (LSD) multiple comparison procedure. For all comparisons *P*<0.05 was considered significant.

## Results

### Effects of MBP on cell viability

**Figure 1:**
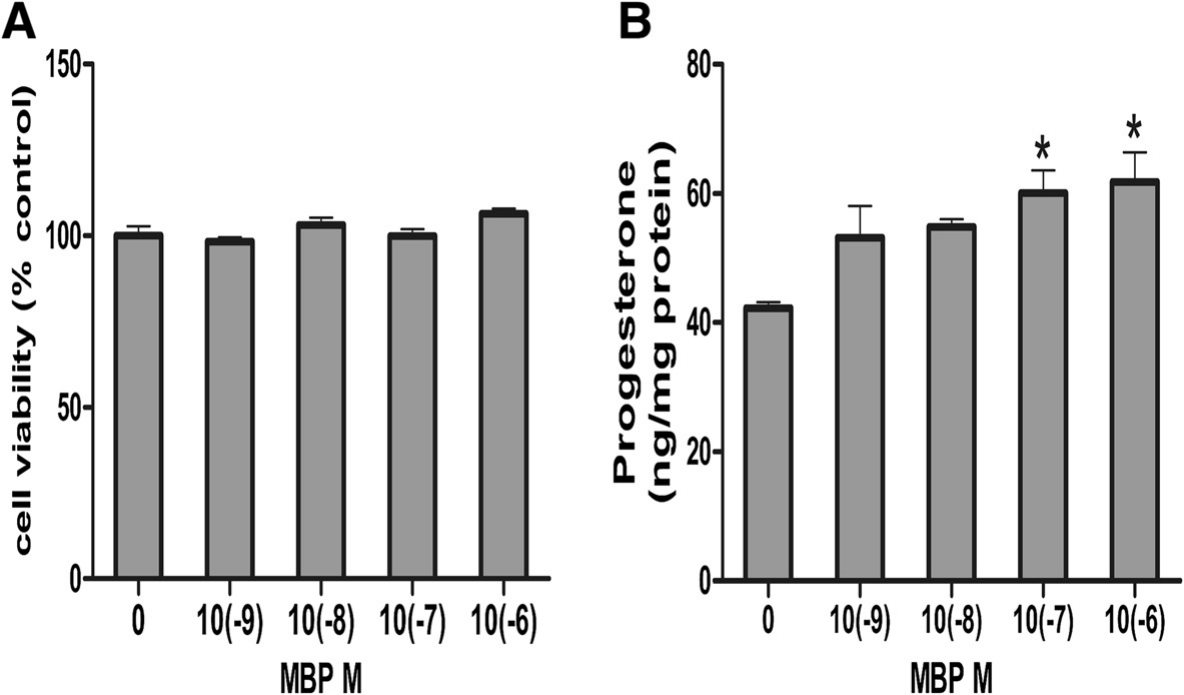
**Analysis of cell viability and production of progesterone in MBP treated MLTC-1 cells. (A)** Cells were cultured with various concentrations of MBP (10(−9), 10(−8), 10(−7), or 10(−6) M) or DMSO as control for 24 h. At the end of incubation, the cell viability was determined using MTT assay. Results were presented as percentage of the values measured in untreated control cells. Each data point represented as mean ± S.E. from three separate experiments in which treatments were performed in quadruplicate. No statistically significant difference was found between the treatment groups and the control group. **(B)** Cells were treated with various concentrations of MBP (10(−9), 10(−8), 10(−7), or 10(−6) M) or DMSO as control for 24 h, and then the medium was changed to SFM and cells were stimulated with hCG (0.1 U/ml) for 4 h. Each data point represents as mean ± S.E. from at least three separate experiments in which treatments were performed in quadruplicates. * indicates significant difference when the values were compared with the control (*P*< 0.05).

Cytotoxicity was not observed following treatments with MBP (10(−9), 10(−8), 10(−7), or 10(−6) M) for 24 h in MLTC-1 cells. Therefore, these doses were selected in the following test. The cell viability result is shown in Figure 1A.

### MBP increased progesterone production

According to the cell viability results, MLTC-1 cells were treated with various doses of MBP for 24 h and then stimulated by hCG (0.1 U/ml) for 4 h *in vitro*. Compared with control group treated by DMSO, production of progesterone was increased after MBP treatments in dose-dependent manner with the increase of 29.76% and 31.72% in 10(−7) and 10(−6) M of MBP, respectively (Figure 1B).

### MBP increased mRNA and protein levels of StAR

**Figure 2:**
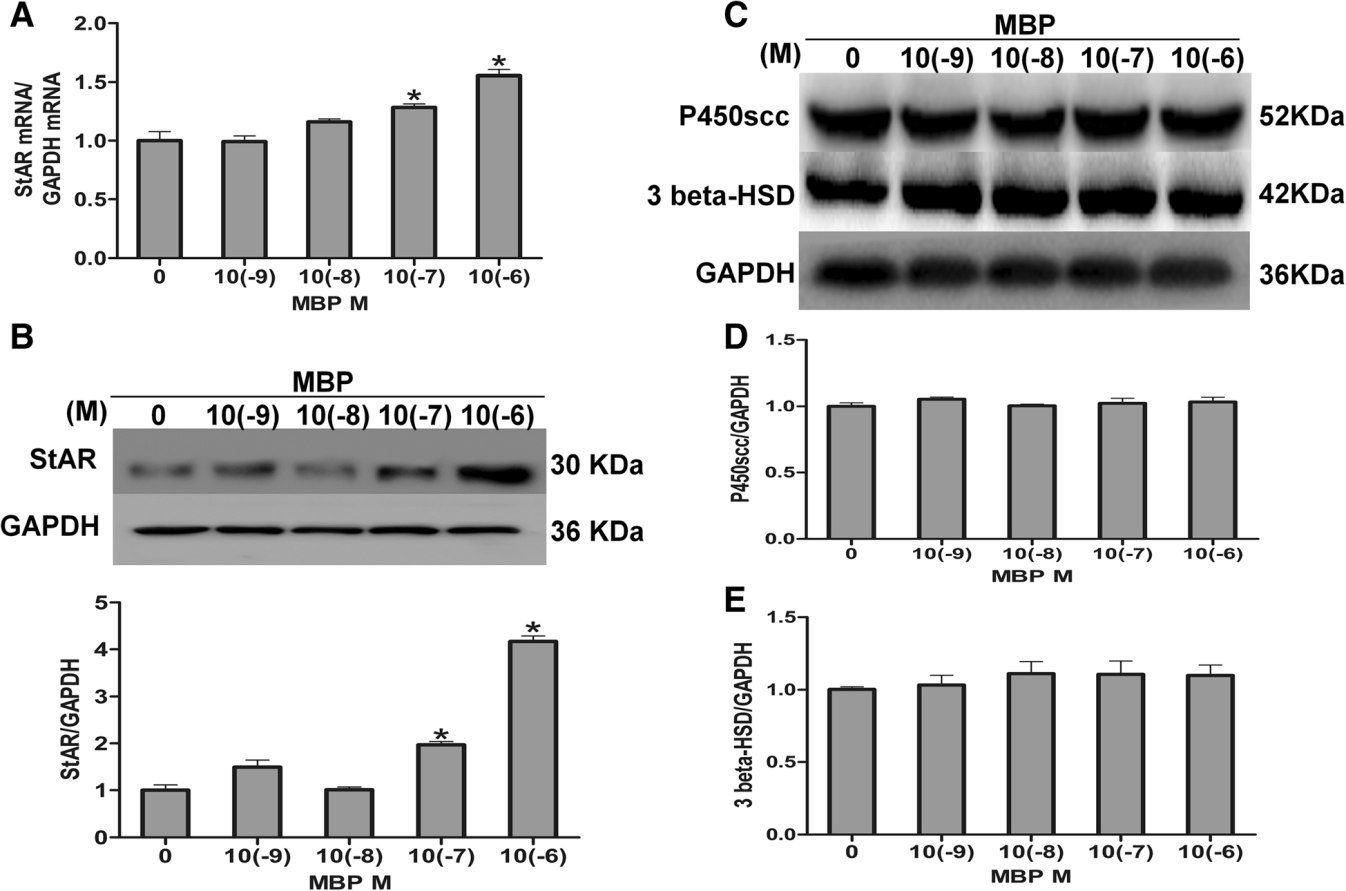
**Expression of StAR, P450scc and 3 beta-HSD in MBP treated MLTC-1 cells.** Cells were treated with MBP (10(−9), 10(−8), 10(−7), or 10(−6)M) or DMSO as control for 24 h and then stimulated with hCG for 4 h. **(A)** StAR mRNA levels were determined by quantitative real-time RT-PCR using GAPDH as housekeeping gene. **(B) **The panel shows a representative western blot analysis of StAR protein expression corrected by corresponding GAPDH. The figure represents as mean ± S.E. of StAR protein integrated optical density corrected by corresponding GAPDH. Each data point was normalized to the control (DMSO) and represents as mean ± S.E. from three independent experiments. * indicates significant difference when the values were compared with the control (*P*< 0.05). **(C**-**E)** The panel shows a representative western blot analysis of P450scc and 3 beta-HSD protein expression corrected by corresponding GAPDH. The figure represents as mean ± S.E. of P450scc and 3 beta-HSD protein integrated optical density corrected by corresponding GAPDH. Each data point was normalized to the control (DMSO) and represents as mean ± S.E. from three independent experiments.

As we found low-dose MBP-stimulated steroidogenesis, we further explored the mechanisms underlying the increase of progesterone production. We examined the expression of genes involved in steroid biosynthesis pathway including StAR, cytochrome P450scc and 3 beta-hydroxy-steroid dehydrogenase (3 beta-HSD) [19,20,27]. Results showed that levels of StAR mRNA and protein were unchanged in both 10(−9) and 10(−8) M but increased markedly after exposure to10 (−7) and 10(−6) M MBP. Compared with control group, MBP increased 1.28-fold and 1.55-fold of mRNA in 10(−7) and 10(−6) M groups, respectively (Figure 2A). Also, MBP increased 1.97-fold and 4.17-fold of protein levels in 10(−7) and 10(−6) M groups, respectively (Figure 2B). However, protein levels of P450scc and 3 beta-HSD were unchanged after MBP exposure (Figure 2C, 2D and 2E). Therefore, our next study focused on the regulation of StAR expression after MBP exposure.

### MBP increased DNA-binding of SF-1, GATA-4 and C/EBP-beta with promoter regions of StAR

**Figure 3:**
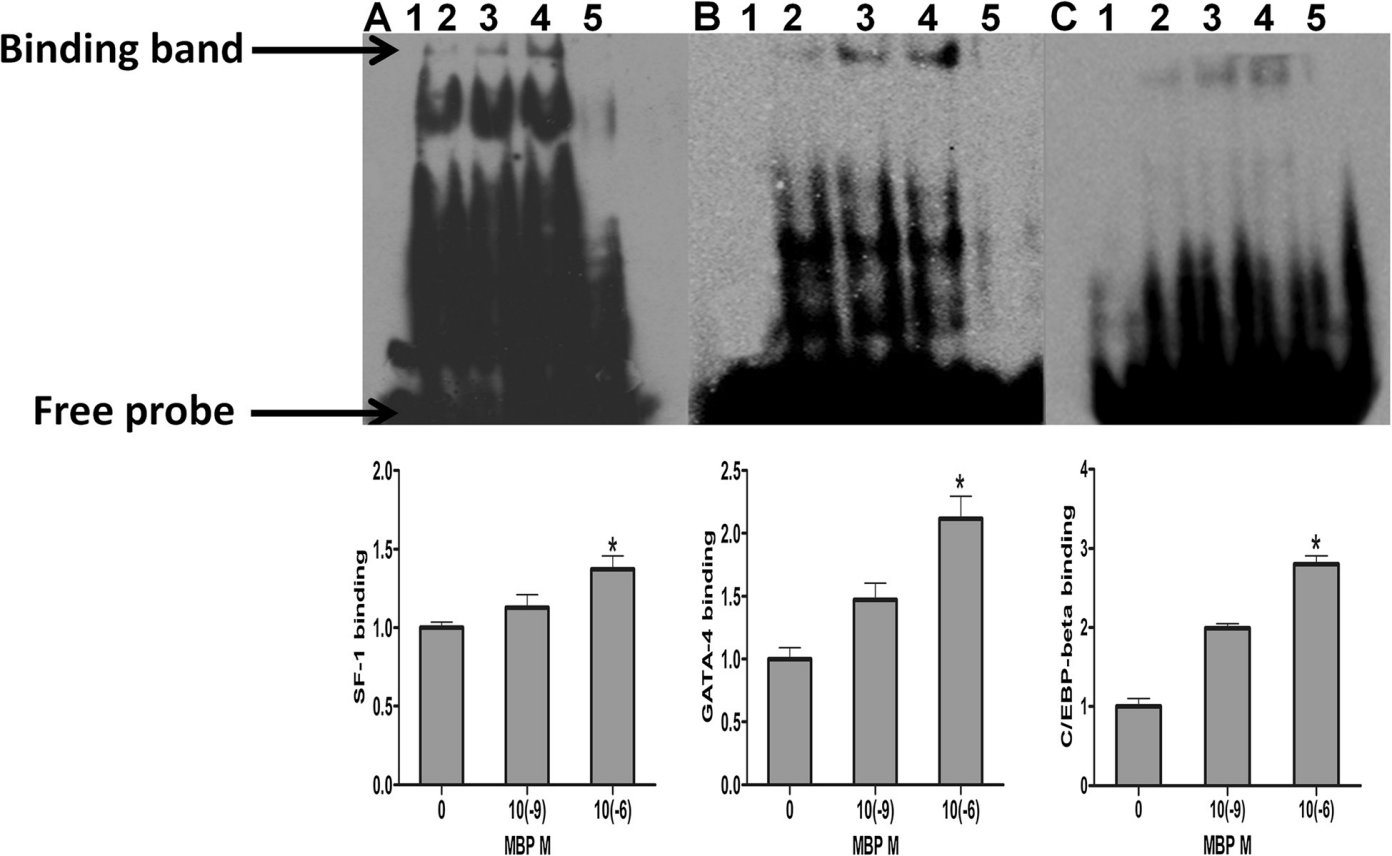
**Examination of transcription factors binding in MBP treated MLTC-1 cells. (A**-**C)** The binding of SF-1, GATA-4, and C/EBP-beta with the StAR promoter regions after MBP treatment for 24 h, and then stimulated with hCG for 4 h. It was evaluated by an EMSA described in the “Materials and Methods” section. Lane 1: blank (only labelled probe); Lane 2: control-without MBP treatment (labelled probe and nuclear protein); Lane 3: treated with 10(−9) M MBP for 24 h (labelled probe and nuclear protein); Lane 4: treated with10 (−6) M MBP for 24 h (labelled probe and nuclear protein); Lane 5: positive control (unlabeled probe, labelled probe, and nuclear protein). The DNA-binding of SF-1, GATA-4 and C/EBP-beta with the StAR promoter regions was determined by quantified bar graph using control without MBP treatments. * indicates significant difference when the values were compared with control (*P*<0.05).

Multiple lines of evidence demonstrated the involvements of several transcription factors including SF-1, GATA-4 and C/EBP-beta in StAR expression and steroidogenesis [26,28]. We further investigated whether MBP-induced StAR expression alteration were resulted from these transcription factors. Interestingly, EMSA results revealed that DNA-binding of SF-1, GATA-4 and C/EBP-beta with promoter regions of StAR was elevated in10(−6) M MBP group (Figure 3A, 3B and 3C), indicating SF-1, GATA-4 and C/EBP-beta might play an important role in low-dose MBP stimulated StAR expression.

### MBP induced increase of protein levels of SF-1, GATA-4, C/EBP-beta and decrease of protein levels of DAX-1

**Figure 4:**
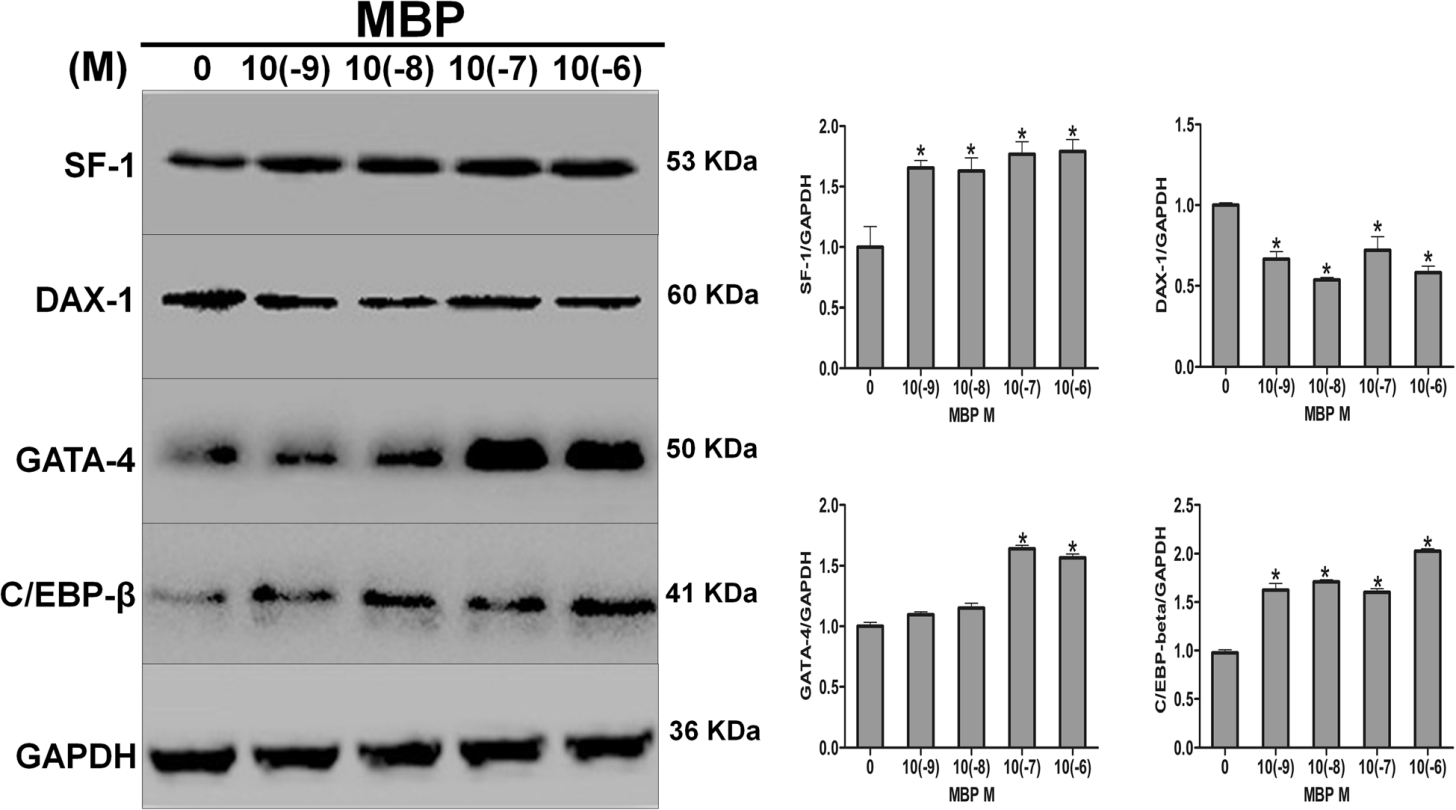
**Expression of transcription factors protein in MBP treated MLTC-1 cells.** Cells were treated with MBP (10(−9), 10(−8), 10(−7), or 10(−6) M) or DMSO as control for 24 h, and then stimulated with hCG for 4 h. The panel shows a representative western blot analysis of SF-1, GATA-4, C/EBP-beta and DAX-1 protein expression corrected by corresponding GAPDH. SF-1, GATA-4, C/EBP-beta and DAX-1 protein expressions were determined by quantified bar graph using GAPDH. * indicates significant difference when the values were compared with the control (*P*< 0.05).

In order to further validate the role of SF-1, GATA-4 and C/EBP-beta, aside from DNA-binding, protein levels of SF-1, GATA-4, and C/EBP-beta were examined using western blot. Protein levels of SF-1, GATA-4 and C/EBP-beta were increased after low-dose MBP treatment (Figure 4). We also analyzed the protein levels of DAX-1(a negative factor of SF-1) after MBP treatment. We found that DAX-1 protein levels were significantly decreased after MBP exposure (Figure 4).

## Discussion

PAEs are considered to be one of endocrine disrupting chemicals (EDCs) [29,30]. From a number of researches, consistent toxicological evidence of adverse reproductive effects of DBP and other phthalates could be found at relatively high doses such as 50, 100, 200, 400 and 800 μM of MBP [31]. In recently published paper, Vandenberg et al. (2012) reviewed mechanistic data for low-dose effects of EDCs, and claimed that EDCs can have effects at low doses that are not predicted by effects at higher doses [32]. Indeed, effects of low-dose chemicals, or called hormesis, have received considerable attention over the past several years [33,34]. In the present study, low-dose effects of MBP on steroidogenesis were examined *in vitro* using MLTC-1 cells as a model. Results demonstrated that steroidogenesis was increased after 10(−7) and 10(−6) M MBP treatments, suggesting that MBP exhibited stimulation effects on steroidogenesis at low doses in MLTC-1 cells. This finding is consistent with previous reports (Fan J, et al. 2010. Endocrinology 151, 3348–62)[18]. In order to further explore the underlying mechanisms, we examined expression of genes involved in steroid biosynthesis pathway, and found mRNA and protein of StAR, a key and rate-limiting protein in steroidogenesis, were elevated by MBP in the similar manner, suggesting that MBP-induced increase of steroidogenesis may result from stimulation of StAR protein expression.

It is well known that StAR expression is modulated by many transcription factors. Previous studies showed that SF-1, GATA-4 and C/EBP-beta may be closely related to the function of steroidogenic cells [35]. Particularly, these factors played important roles in regulation of StAR expression [36]. Therefore, transcription factors may be involved in the alteration of StAR mRNA and protein levels after MBP treatments.

SF-1 is a nuclear receptor that plays multiple roles throughout the hypothalamic-pituitary-steroidogenic organ axis, which has been characterized as a master regulator of steroidogenesis and can function at StAR gene promoter regions to control StAR transcription [37-39]. Also, it has been found that mutation in the SF-1 binding sites resulted in decreased StAR expression in *in vitro* reporter gene assays [24,40], and it has been reported that SF-1 knockout mice did not express StAR mRNA, indicating SF-1 is required for StAR gene expression [41]. Thus, SF-1 may play an important role in positive regulation of StAR expression. Previous studies have shown that bind sites of SF-1 to StAR promoters in rat, humans, porcine, ovine and mouse, were at −493/-483, -456/-445, -143/-132, -106/-97, -764/-754 and −46/-40 [24,42-44]. In our study, we detected DNA-binding of SF-1 at −46/-40 of StAR promoters. We found that DNA-binding of SF-1 was increased by MBP treatment at 10(−6) M (Figure 3A). At the same time, SF-1 protein expression was elevated after exposure to MBP (Figure 4). Moreover, *in vitro* studies showed that DAX-1 could inhibit SF-1-mediated transactivation [45]. Therefore, to further validate the SF-1 role in MBP-stimulated StAR expression, DAX-1 protein levels were next analyzed by western blot. We found that DAX-1 protein expression was lowed in dose-dependent manner (Figure 4). Collectively, increased progesterone production by exposure to MBP may be regulated by SF-1 and subsequently affecting StAR expression. Thus, it is likely that one of the important mechanisms of low-dose MBP-induced stimulation of steroidogenesis may be mediated by SF-1 regulation on StAR.

GATA-4, a member of GATA group of conserved transcriptional regulators with a highly conserved zinc finger DNA-binding domain, is expressed in steroidogenic cells [46,47], and regulates gene expression, differentiation and cell proliferation by binding to the consensus DNA sequence [48]. At the same time, GATA-4 is important for activation of StAR transcription by binding to the proximal *cis*-elements in the StAR promoter [49]. Previous studies revealed a GATA binding element at position −66/−61 in the mouse StAR promoter [25,50]. In this study, we found that DNA-binding of GATA-4 was increased at 10(−6) M after MBP treatments (Figure 3B). Also, GATA-4 protein expression was increased in dose-dependent manner (Figure 4). Taken together, GATA-4 may also undertake in steroidogenesis after low-dose MBP treatments.

C/EBP-beta presents in steroidogenic cells including Leydig and granulosa cells [51], which plays a vital role in LH-regulated Leydig cell differentiation and function. C/EBP-beta is regulated by hCG/LH [52]. It was reported that C/EBP-beta represented the ability of binding with StAR promoter regions in rats [36,53]. Moreover, related study indicates C/EBP-beta can positively regulate basal StAR gene expression [54]. In the present study, oligonucleotide sequences at −117/-108 were selected for C/EBP-beta binding with StAR promoters [23]. We found that C/EBP-beta could bind with StAR promoter regions and DNA-binding of C/EBP-beta was increased after MBP exposure (Figure 3C). These findings agree with previous report in which 8-Br-cAMP stimulation also altered this molecular event [36]. Also, C/EBP-beta protein expression was also increased after low-dose MBP treatments (Figure 4). Thus, aside from SF-1 and GATA-4, the increased DNA-binding and expressions of C/EBP-beta may also result in the elevation of StAR protein and production of progesterone by low-dose MBP treatments.

## Conclusions

**Figure 5:**
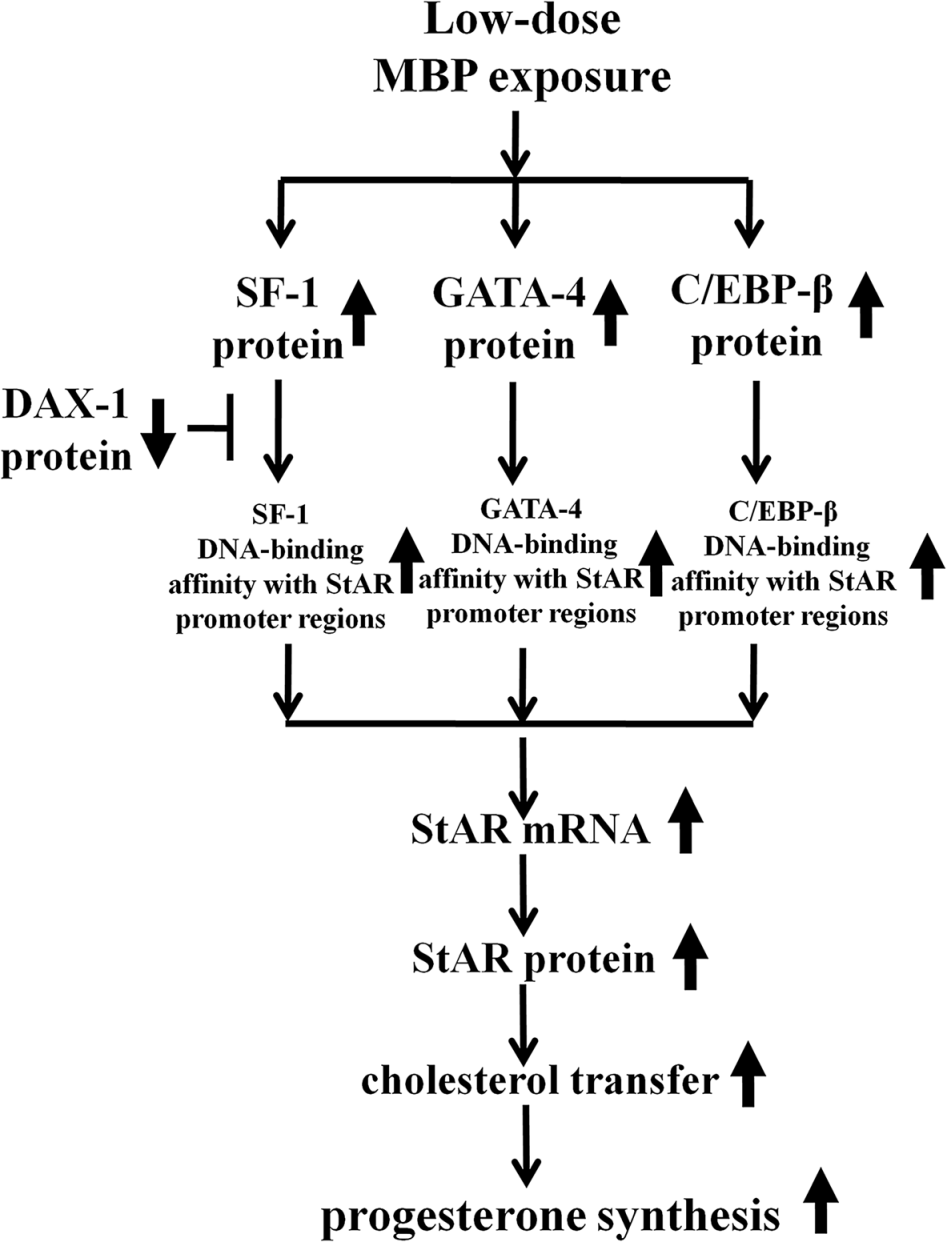
Schematic presentation of low-dose MBP-stimulated progesterone synthesis in MLTC-1 cells.

In summary, we firstly delineated that regulation of StAR by transcription factors including SF-1, GATA-4 and C/EBP-beta maybe critical mechanisms involved in low-dose MBP-stimulated steroidogenesis. Low-dose MBP may alter DNA-binding of SF-1, GATA-4, C/EBP-beta and expression of SF-1, GATA-4, C/EBP-beta protein, and then stimulates transcription and expression of StAR. Besides, MBP-induced downexpression of DAX-1 which is the negative factor of SF-1 may also be involved in this process. Following these molecule events, steroid production was stimulated at last (Figure 5). Herein, we provide a new horizon in low-dose effects associated with transcription factors by MBP exposure. Our data should promote further study on providing deep insight into the initial molecular actions which influence SF-1, GATA-4, and C/EBP-beta in PAEs-stimulated steroidogenesis.

## Abbreviations

C/EBP-beta: CCAAT/enhancer binding protein-beta; DBP: Dibutyl phthalate; EMSA: Electrophoretic mobility shift assay; hCG: Human chorionic gonadotropin; MBP: Monobutyl phthalate; MLTC-1: Mouse Leydig tumor cells-1; MTT: -(4,5)-dimethylthiahiazo(−z-y1) -3,5-di-phenytetrazoliumromide; PAEs: Phthalate esters; RIA: Radioimmunoassay; RT-PCR: Real-time reverse transcription polymerase chain reaction; SF-1: Steroidogenic factor 1; StAR: Steroidogenic acute regulatory protein.

## Competing interests

The authors declare that they have no competing interests.

## Authors’ contributions

Conceived and designed the experiments: YH YW. Performed the experiments: YH CD MC YC LQ. Analyzed the data: YH CD MC JL XH JQ XL. Contributed reagents/materials/analysis tools: AG YK HS ZL. Wrote the paper: YH. All authors read and approved the final manuscript.
